# Pre-operative proton versus photon-based chemoradiotherapy as an addition to best systemic therapy in the management of oesophageal cancer: protocol for the UK multi-centre randomised phase 2 PROTIEUS study

**DOI:** 10.1186/s12885-026-16424-1

**Published:** 2026-06-29

**Authors:** Ganesh Radhakrishna, Christopher M. Jones, C. William Bleaney, Jillian Blackshaw, Natalie Blencowe, Douglas Brand, Caroline S. Clarke, Sarah Gwynne, Natasha Hava, Anna King, Geraint John Lewis, Andre Lopes, Ka Man Mak, Elizabeth Miles, Somnath Mukherjee, Owen Nicholas, Memuna Rashid, Kasia Owczarczyk, Kai-Keen Shiu, Elizabeth Smyth, John Tattum, Tim Underwood, Catarina Veiga, Maria A. Hawkins

**Affiliations:** 1 The Christie Hospital, The Christie Hospitals NHS Foundation Trust, Wilmslow Road, Manchester, M20 4BX UK; 2 Division of Cancer Sciences, University of Manchester, Manchester, UK; 3 Department of Oncology, University of Cambridge, Cambridge, UK; 4 Addenbrooke’s Hospital, Cambridge University Hospitals NHS Foundation Trust, Cambridge, UK; 5 Patient Advocate, Warrington, UK; 6 University of Bristol, Bristol, UK; 7 Department of Medical Physics & Biomedical Engineering, University College London, London, WC1E 6BT UK; 8 Research Department of Primary Care and Population Health, University College London, London, UK; 9 South West Wales Cancer Centre, Swansea, UK; 10 National Radiotherapy Trials QA (RTTQA) Group, East and North Hertfordshire Teaching NHS Trust, Mount Vernon Cancer Centre, London, UK; 11 Cancer Research UK & UCL Cancer Trial Centre, University College London, London, UK; 12 Oxford University Hospitals NHS Foundation Trust, Oxford, UK; 13 Department of radiotherapy, Guys and St Thomas Hospital, London, UK; 14 Velindre Cancer Centre, Velindre University NHS Trust, Cardiff, UK; 15 University College London Hospital, London, UK; 16 Patient Advocate, Wigan, UK; 17 University of Southampton, Southampton, UK

**Keywords:** Chemoradiotherapy, Oesophageal adenocarcinoma, Oesophageal squamous cell carcinoma, Proton beam therapy, Radiotherapy, Survival, Tolerability, Toxicity

## Abstract

**Background:**

Oesophageal cancer is a major cause of morbidity and mortality. Around 40% of patients present with locally advanced disease. Outcomes are poor, with 5-year survival of around 50% for locally advanced oesophageal adenocarcinoma (OAC) and 60% for oesophageal squamous cell carcinoma (OSCC). Over a fifth of recurrences are locoregional. These may be reduced by the addition of chemoradiotherapy (CRT) to best pre-operative systemic anti-cancer therapy (SACT). However, photon-based CRT is associated with a higher frequency of complications than chemotherapy alone. This study will explore whether, in patients with locally advanced OAC and OSCC managed with hypofractionated CRT following best pre-operative systemic therapy, the use of proton beam therapy (PBT) reduces post-operative complications when compared with photon-based treatment.

**Methods/design:**

PROTIEUS is an investigator-initiated randomised multi-centre phase 2 trial aiming to recruit 170 patients with locally advanced OAC (*n* = 130) or OSCC (*n* = 40) from the UK National Health Service. Patients with locally advanced cT ≥ 2, N0-2 non-metastatic disease are eligible for inclusion and will be randomised 1:1 to receive 40.05 Gy (RBE) in 15 fractions over three weeks using either PBT or intensity modulated/rotational arc photon radiotherapy. All patients will receive three one-week cycles of concurrent intravenous carboplatin/paclitaxel. CRT will be delivered following best pre-operative SACT. The primary endpoint is the rate of severe post-operative complications within 90 days post-surgery (grade 3 or higher Clavien-Dindo classification and CTCAE v5.0). Secondary endpoints relate to efficacy (pathological complete response and clear resection margin rate, disease-free and overall survival), tolerability (completion of planned CRT regime, time to and completion of adjuvant therapy), morbidity (long-term quality of life measures) and health economics (cost-effectiveness analyses using EQ-5D-5 L and resource use assessments).

**Discussion:**

There is a need to improve local control in OAC and OSCC. Given only modest gains with perioperative immune checkpoint inhibition and a disease landscape in which there are few targets for precision or personalised therapies, there are limited options to achieve further gains across the disease population using SACT. This study will therefore determine which of PBT or photon-based pre-operative CRT is the most tolerable and least toxic for integration into existing systemic treatment paradigms.

**Trial registration:**

https://doi.org/10.1186/ISRCTN50098578.

**Supplementary Information:**

The online version contains supplementary material available at https://doi.org/10.1186/s12885-026-16424-1.

## Background

Oesophageal cancer is a major cause of morbidity and mortality, accounting annually for 445,00 deaths and the loss of 9.8 million disease-adjusted life years [1, 2]. Between 85 and 90% of cases worldwide are oesophageal squamous cell carcinoma (OSCC) [3]. However, the incidence of oesophageal adenocarcinoma (OAC) has increased markedly since the 1970s and it is now the predominant subtype in over 20 high-income countries, including the United Kingdom (UK) [3].

Around 40% of patients newly presenting with oesophageal cancer have locally advanced disease [4]. Management strategies for these patients vary by histological subtype. For patients with OAC, the ESOPEC trial has, since its publication in 2025, established surgical resection with perioperative 5-fluorouracil with leucovorin, oxaliplatin and docetaxel (FLOT) as the standard of care for most patients [5, 6]. By contrast, options for curative-intent treatment for locally advanced OSCC are more nuanced and standards of care vary. The CROSS trial established a 5-year survival of 61% following neoadjuvant carboplatin and paclitaxel-based chemoradiotherapy (CRT) followed by resection [7]. Outcomes are improved with the addition of adjuvant immune checkpoint inhibition (ICI) using nivolumab for patients for whom CRT does not achieve a pathological complete response (pCR) [8]. Definitive CRT with close surveillance and salvage resection, as required, forms an alternative standard of care [9].

Outcomes are poor for locally advanced OAC and OSCC. In the ESOPEC trial, perioperative FLOT delivered pCR in only 16.7% of patients with OAC [5]. This translated to a 5-year overall survival (OS) of 50.6%^5^. A subsequent analysis of recurrence patterns for patients managed with perioperative FLOT has demonstrated a 3-year cumulative incidence of locoregional and distant recurrence of 20.2% and 31.5%, respectively [10]. Around a third of distant recurrences occur with simultaneous locoregional recurrence [10]. The frequency of recurrence may be modestly reduced by perioperative ICI, though supporting data from the MATTERHORN trial are drawn from a population of patients with gastric and gastroesophageal junctional (GOJ) adenocarcinoma [11]. In this study, the addition of durvalumab to FLOT resulted in a significant improvement in two-year event-free survival (67.4% vs. 58.5%; HR 0.71, 95% confidence interval, CI 0.58–0.86). A 5.3% point (75.7% vs. 70.4%) increase in overall survival was also observed at the same time interval, albeit without reaching a pre-defined threshold for significance [11]. However, whilst pCR was higher with durvalumab (19.2% vs. 7.2%, relative risk 2.69 (95%CI 1.86–3.90)), R0 resection rates were similar (91.5% vs. 92.3% for placebo) [11].

Consequently, there remains a need to enhance both local and systemic disease control in patients with locally advanced OAC. A key challenge is the considerable inter- and intra- tumoural heterogeneity that dominates OAC, resulting in a landscape with few actionable targets for new precision and personalised treatment approaches [12]. However, the TOPGEAR trial highlighted that the addition of CRT to cytotoxic chemotherapy is safe and results in a close to doubling of pCR (17% vs. 8%), a substantial improvement in major pathological response (50% vs. 29%) and greater tumour downstaging (T1/T2 32% vs. 25%) with higher R0 rates (92% vs. 88%) in patients with gastric or GOJ adenocarcinomas [13, 14]. Further, whilst the addition of CRT to best systemic therapy did not improve overall survival across the predominantly gastric cancer population enrolled in TOPGEAR, subgroup analyses demonstrated a benefit from CRT in the subgroup of patients with tumours arising from the GOJ. Early data from the CRITICS-II trial also support the safety and efficacy of the addition to SACT of CRT, albeit again in the context of gastric cancer [15]. In this multi-centre phase 2 study, the addition of CRT following triplet chemotherapy (docetaxel, oxaliplatin and capecitabine) improved 1-year event-free (84% vs. 68%) and overall (89% vs. 74%) survival versus chemotherapy alone. This sequential approach also resulted in improved event-free (84% vs. 78%) and overall (89% vs. 84%) survival when compared with CRT [15].

These data together point to activity of CRT in cellular populations that otherwise demonstrate resistance to systemic therapy. Given this, there is rationale for the addition of CRT to pre-operative FLOT. However, use of photon-based neoadjuvant CRT is associated with a higher rate of 90-day post-operative complications versus chemotherapy alone (65% vs. 59%; *p* < 0.001) [16]. Delivering 41.4 Gy in 23 fractions over 4.5 weeks, the conventional CROSS-style neoadjuvant CRT regimen also places a substantial burden on patients who have already received eight weeks of treatment using FLOT, diminishing the potential advantage of adding CRT to conventional chemotherapy regimens. We have previously shown that shorter, hypofractionated (i.e. >2.4 Gy per fraction) radiotherapy regimens are safe and tolerable in patients with oesophageal cancer [17]. Others have also highlighted a low α/β ratio that may favour increased efficacy with use of hypofractionated techniques [18]. Moreover, early phase studies have shown promising results for proton beam therapy (PBT), which when used for oesophageal cancers is associated with reduced cardiac and lung toxicity[19] and reduced rates of grade 4 lymphopenia [20–22].

Since these data apply across oesophageal cancer subtypes, there is merit in evaluating the efficacy of hypofractionated radiotherapy in patients with OAC and OSCC. It is not known whether PBT delivers improved toxicity when used as part of a hypofractionated regimen. Consequently, there is a need for a phase 2 trial in which the toxicity and tolerability of photon versus PBT based hypofractionated radiotherapy is assessed in patients with OAC and OSCC when used as an adjunct to best peri-operative systemic therapy.

## Trial design /methods

### Aim

The PROTIEUS trial will assess whether neoadjuvant PBT-based hypofractionated CRT significantly reduces the risk of grade 3 or above post-operative complications within 90 days of surgery compared with photon-based hypofractionated CRT, when used sequentially with best systemic therapy in patients with locally advanced OAC and OSCC.

### Design & setting

**Fig. 1:**
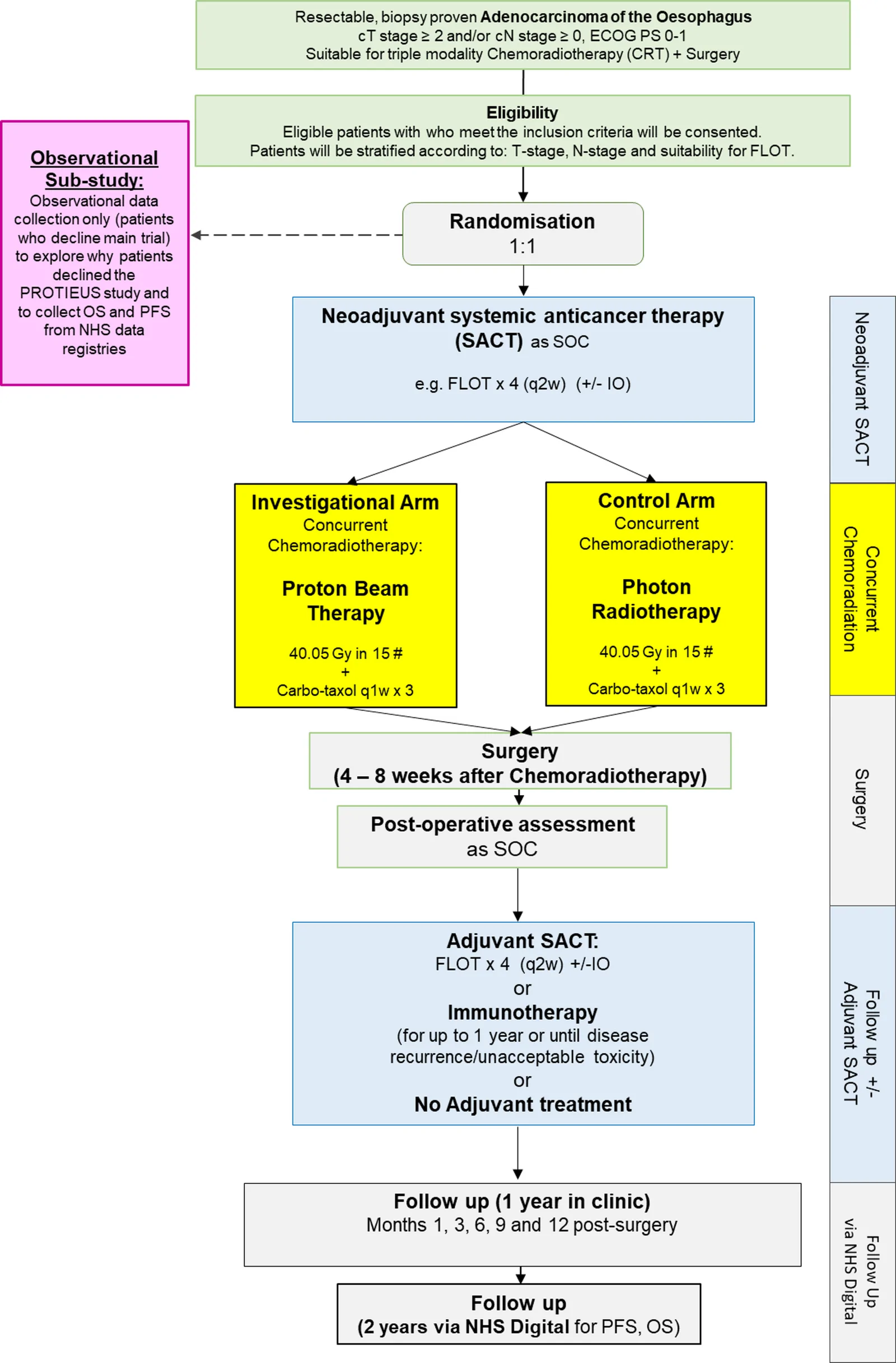
An overview of the PROTIEUS trial approach for patients with oesophageal adenocarcinoma. Carbo-taxol: carboplatin, paclitaxel; CRT: chemoradiotherapy; ECOG PS: Eastern Cooperative Oncology Group performance status; FLOT: 5-fluorouracil, leucovorin, oxaliplatin, docetaxel; IO: immunotherapy; OS: overall survival; PFS: progression-free survival; q2w: 2-weekly; SACT: systemic anti-cancer therapy; SOC: standard of care

**Fig. 2:**
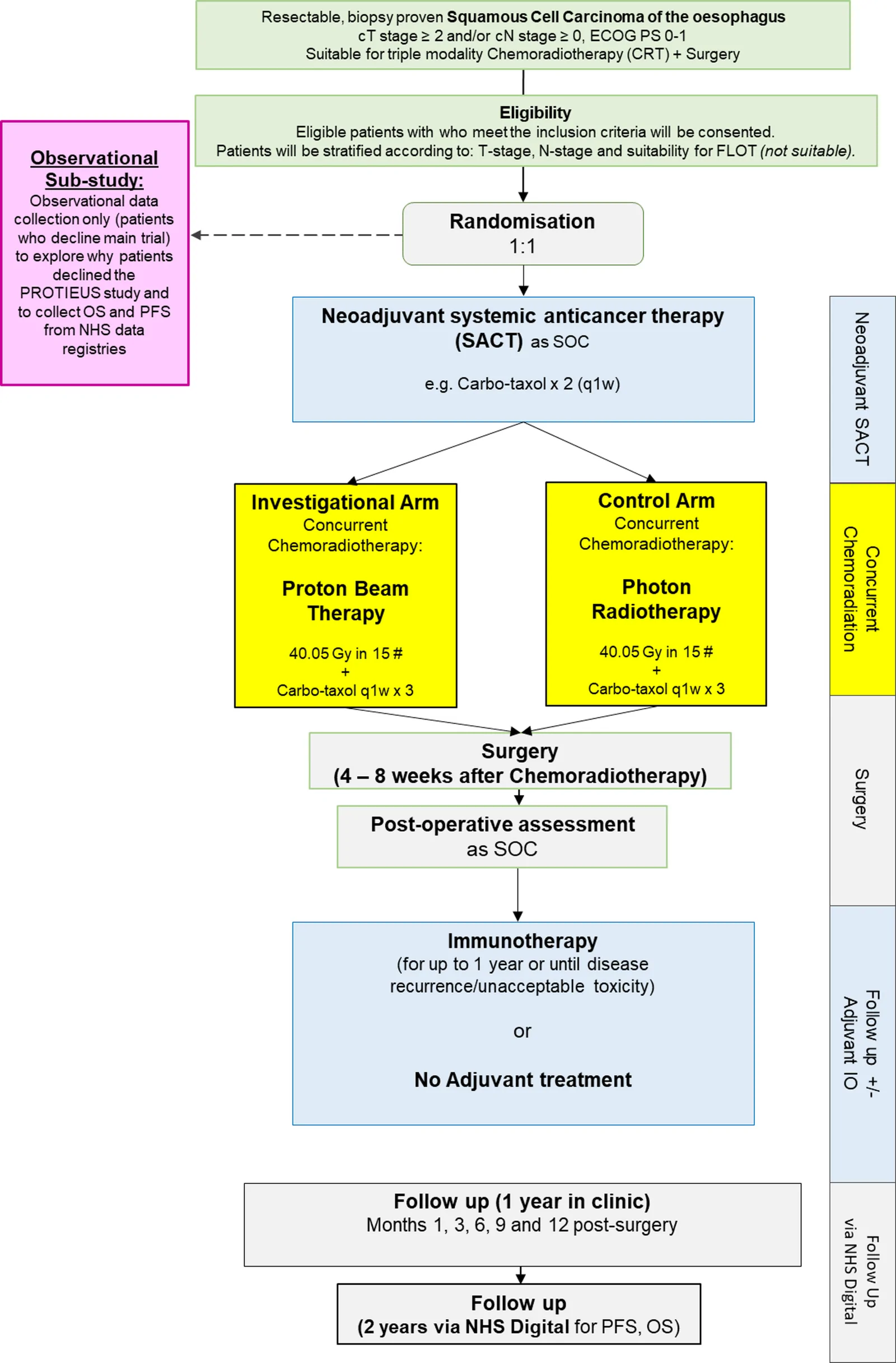
An overview of the trial approach for patients with oesophageal squamous cell carcinoma. Carbo-taxol: carboplatin, paclitaxel; CRT: chemoradiotherapy; ECOG PS: Eastern Cooperative Oncology Group performance status; FLOT: 5-fluorouracil, leucovorin, oxaliplatin, docetaxel; IO: immunotherapy; OS: overall survival; PFS: progression-free survival; q2w: 2-weekly; SACT: systemic anti-cancer therapy; SOC: standard of care

This is an investigator-initiated UK multi-centre, open-label randomised phase 2 trial. An overview of the study approach for OAC and OSCC is provided in Figs. 1 and 2. The planned sample size is described below. All patients will be managed within the National Health Service (NHS) using 40.05 Gy (relative biological effectiveness, RBE) in 15 fractions radiotherapy over three weeks with three cycles of concurrent weekly intravenous carboplatin/paclitaxel. PBT will be administered at one of two centralised centres (University College London Hospitals NHS Foundation Trust, London, or The Christie Hospitals NHS Foundation Trust, Manchester). Photon-based radiotherapy will be delivered in local tertiary referral centres using either intensity modulated (IMRT) or rotational arc (VMAT) radiotherapy. For patients with OAC, CRT will be delivered after patients have received up to four cycles of pre-operative FLOT, with or without a concurrent immunotherapy agent. Patients with OSCC will receive two intravenous once weekly cycles of carboplatin/paclitaxel prior to CRT. Following CRT, patients will proceed to standard surgical resection and will then receive adjuvant therapy in line with usual local practice and investigator choice. Adjuvant options will include four cycles of FLOT, with or without an immunotherapy agent in OAC, and the option for single-agent nivolumab for patients for whom pre-operative treatment did not result in pCR (as determined by the treating site) in OSCC and OAC. The PI, or, where delegated by the PI, other appropriately trained site staff, are required to provide a full explanation of the trial and all relevant treatment options to each patient prior to trial entry. During these discussions, the current approved patient information sheets for the trial should be discussed with the patient Those patients who do not consent to participate in the main trial will be invited to take part in an observational sub-study, within which around 65 patients’ rationale for not pursuing trial participation will be explored.

The Trial Management Group (TMG) will include the Chief Investigator, clinicians, patients and experts from relevant specialties and PROTIEUS trial staff from UCL CTC (see page 1). The TMG will be responsible for overseeing the trial. The group will meet regularly, at least twice a year, and will send updates to PIs (via newsletters or at Investigator meetings).

The TMG will review substantial amendments to the protocol prior to submission to the REC. All PIs will be kept informed of substantial amendments through their nominated responsible individual and are responsible for their prompt implementation This study has been co-designed and supported by patients on the TMG.

The study will be subject to safety monitoring from an Independent Data Monitoring Committee (IDMC) and will be overseen by a dedicated TMG. The IDMC will meet at least annually, at which point conditional power for the primary endpoint will be presented to assess the probability of achieving statistical significance at the end of the trial, conditional on the interim data and assuming that the current trend continues.

### Target population

Eligible patients aged 16 years and over will have a histologically-confirmed diagnosis of locally advanced, non-metastatic OAC or OSCC affecting the thoracic oesophagus or GOJ. All patients must provide written informed consent and all must be considered by a specialist multidisciplinary team to be fit for neoadjuvant chemotherapy and CRT followed by surgery. The following additional inclusion criteria will apply:

1. cT stage ≥ 2 and/or cN stage 0–2, as defined by the eighth edition of the American Joint Committee on Cancer staging system [23].
2. Eastern Cooperative Oncology Group (ECOG) performance status 0–1.
3. Adequate cardiovascular and respiratory function for surgery as determined by the surgical team within a four-week period prior to randomisation.
4. Willing and able to receive treatment at a PBT centre in London or Manchester.

Patients must also not meet any of the following exclusion criteria:

1. Tumour extends into the stomach by more than 3 cm beyond the GOJ.
2. The presence of extensive nodal disease (N3) or metastatic disease.
3. Patients who have previously received treatment for invasive oesophageal or GOJ, including with photodynamic therapy or laser therapy for high grade dysplasia or carcinoma in situ.
4. A tumour length of > 10 cm in the thorax or > 12 cm at the GOJ or in the lower third of the oesophagus, or a total length of disease (encompassing the primary tumour and involved lymph nodes) of > 14 cm for lower third tumours.
5. Patients with an oesophageal stent.
6. Patients with significant cardiac disease, including unstable angina, uncontrolled hypertension, cardiac failure or arrhythmia.
7. Patients with significant respiratory disease, including usual interstitial pneumonia, another form of interstitial lung disease or a connective tissue disorder.
8. History of malignancy likely to interfere with the protocol treatment (i.e. patients with previously treated malignancy who have been disease-free for less than 1 year, or those still on active treatment), which does not include subjects successfully treated and disease-free for 3 years, a history of treated non-melanoma skin cancer, successfully treated in situ carcinoma, chronic lymphocytic leukaemia in stable remission, or indolent prostate cancer requiring no, or only anti-hormonal, therapy.
9. Women who are pregnant or breastfeeding.
10. Patients unable to adhere to contraception guidance.
11. Any other situation deemed by the local Principal Investigator to render the patient unsuitable for the trial.

### Screening and pre-treatment assessments

Patients are recommended to undergo endoscopic ultrasound assessment up to six weeks prior to randomisation, though this is not mandated. A computed tomography (CT) or positron emission tomography (PET)-CT must be completed. At least one of these scans should be within 4–6 weeks prior to randomisation. Patients must also undergo lung function tests (cardiopulmonary exercise testing (CPEX) or spirometry) up to 12 weeks prior to randomisation. Additional work-up, to be completed in the seven days prior to randomisation, includes a complete medical history and full physical examination, vital signs, Mellow-Pinkas dysphagia score, ECOG performance status measurement, standard laboratory tests and a pregnancy test. Dihydropyrimidine dehydrogenase testing is also required with fluoropyrimidine doses adjusted in line with local guidance.

### Randomisation

Randomisation will be performed centrally by authorised site staff using the UCL CTC secure web-based OpenClinica system, after written informed consent, confirmation of eligibility and completion of all mandatory pre-randomisation assessments. Participants will be allocated 1:1 to proton beam therapy or photon-based radiotherapy using minimisation with a probabilistic element. Allocation will favour the arm that minimises imbalance across the prespecified factors in 90% of cases, with allocation to the alternative arm in 10% of cases to preserve unpredictability.

The minimisation factors will be clinical T stage (T2,T3, T4), clinical N stage (N0, N1, N2) and FLOT suitability (yes, no). Treatment allocation will be generated and revealed only after eligibility data have been submitted and the randomisation process has been completed, ensuring allocation concealment before assignment. The trial is open-label because radiotherapy modality cannot be masked to participants, treating clinicians or radiotherapy teams.

Patients who meet the eligibility criteria but decline to participate in the main trial will be offered the participant information sheet and informed consent form for the observational sub-study. This sub-study will explore reasons for non-participation and enable follow-up for survival outcomes through routine data registries (NHS Digital). Registration to the observational sub-study will be undertaken by sites using a remote data capture system hosted by UCL CTC.

### Study interventions & quality assurance

Standard-of-care intervention: systemic anti-cancer therapy.

All pre- and post-operative cytotoxic chemotherapy and ICI will be delivered in line with local protocols.

Randomised intervention: proton- versus photon-CRT.

All patients will receive 40.05 Gy in 15 fractions external beam photon- or PBT-radiotherapy over 3 weeks with 3 concurrent 1-week cycles of carboplatin (AUC 2) and paclitaxel (50 mg per m^2^). CRT will commence up to 4 weeks after the final cycle of FLOT, or in the week after completing induction carboplatin/paclitaxel. Radiotherapy will ideally commence on a Monday or Tuesday, with treatment provided on consecutive weekdays. Example treatment schedules for proton- and photon- based treatment in the context of OAC and OSCC are provided in Supplementary File 1.

Following randomisation, and up to 4 weeks prior to commencing radiotherapy, a planning CT scan will be performed. A 4DCT with no greater than 3 mm slice thickness and intravenous contrast (providing renal function permits) in the treatment position is mandated in all cases. Target volume definition will be identical regardless of whether photons or PBT are to be administered and is based on a specific PROTIEUS Radiotherapy Planning Guidance Document (Supplementary File 2). A single-phase inverse-planned IMRT/VMAT plan will be produced for all patients randomised to photons. PBT planning will be performed by physicists in one of the two specialist proton centres. All patients, in both arms. will have daily cone beam CT image verification on treatment with online correction.

Radiotherapy quality assurance will be conducted by the UK National RT Trials Quality Assurance (RTTQA) Group, to include pretrial and on-trial case review. Benchmarking cases will be completed by all participating sites, with prospective review of the first cases and retrospective review of subsequent cases. RT dosimetry and treatment data will be collected for all participants.

Standard-of-care intervention: surgery.

Patients will proceed to surgical resection within 4–8 weeks of completing CRT. Surgery will be performed according to participating surgeons’ preferences, within the following boundaries. Minimally invasive or open 2-phase, 3-phase and thoracoabdominal phases are permitted. Use of a transhiatal approach is prohibited. Lymphadenectomies along the common hepatic artery, left gastric and splenic artery (*en bloc* or separately) will be performed, and the pericardial fat pad will be removed. Crural fibres and a cuff of diaphragm will be removed if required for tumour clearance. Para-oesophageal and diaphragmatic nodes will be removed in continuity with the oesophagus. Lymph nodes at the tracheal bifurcation and along the left and right main bronchi to the pulmonary hilus will be removed (*en bloc* or separately), and strips of pleura, will be removed at surgeons’ discretion. Intra-operative photographs of these key operative steps will be collected to support surgical quality assurance.

### Assessments & monitoring during treatment

A summary of assessments prior to, during and following treatment is provided in Supplementary File 3. Clinical assessment will be performed 2-weekly for patients managed with FLOT and weekly for patients receiving carboplatin/paclitaxel, with bloods taken up to 3 days prior to each cycle of treatment. Assessments will be weekly during CRT with a further assessment within 4–6 weeks of completion of the last fraction of radiotherapy. At each assessment a review of adverse events (AEs) and physical examination will be performed. Performance status and Mellow-Pinkas dysphagia score will also be assessed, as will measurements of ECOG performance status, weight and standard biochemistry plus haematological blood tests. Patients will also undergo a contrast-enhanced CT and, where judged by a site-specialist MDT to be required, a PET-CT within 4–6 weeks of completion of CRT. Repeat pulmonary function and cardiac testing is also recommended at this timepoint, and patients will complete additional quality of life (QoL) questionnaires and a resource use questionnaire.

Surgery will be performed as per standard of care. However, intra-operative photographs will be collected to support surgical quality assurance. Post-operative on-treatment assessments will continue whilst patients receive adjuvant therapy and are in line with those undertaken in the pre-operative period.

### Follow-up assessments

Follow-up assessments are planned for 1, 3-, 6-, 9- and 12-months following surgery. Subsequent survival data will be garnered from available NHS registries. At each follow-up point, an assessment of adverse events (including assessment of post-operative complications using the Clavien-Dindo classification) will be performed, as will a targeted physical examination and measurement of vital signs, ECOG performance status, electrocardiogram (ECG), routine biochemistry and haematological bloods, circulating tumour DNA, QoL measures and participant-completed resource-use questionnaire. A pulmonary function test is also advised but is not mandated. Additional assessments include contrast-enhanced CT imaging at 3, 6 and 12 months. An additional contrast-enhanced CT should be performed 28–35 days after the last administration of adjuvant therapy and at disease recurrence. A further assessment of adverse events and QoL measures will be performed where disease recurs.

The end of the trial will be 24 months after the last patient has completed their final follow up.

### Endpoints

The primary endpoint is the proportion of patients in each treatment arm with one or more severe post-operative complications within 90 days of surgery. Severe complications will be defined as grade 3 or above by Clavien-Dindo [24] and Common Terminology Criteria for Adverse Events (CTCAE) version 5.0 criteria, in line with the International Consensus on Standardisation of Data Collection for Complications Associated with Esophagectomy consensus guidelines [25].

Secondary efficacy endpoints will include pathological complete response, R0 resection, disease-free survival, overall survival and disease recurrence mapping. Pathological complete response will be defined as absence of residual viable cancer in the primary tumour and sampled lymph nodes. R0 resection will be defined as no tumour within 1 mm of any resection margin. Disease-free survival will be defined as the time from randomisation to disease recurrence, progression or death from any cause, whichever occurs first. Overall survival will be defined as the time from randomisation to death from any cause. Disease recurrence mapping will describe the site and pattern of recurrence, including local, locoregional, oligometastatic, widespread, micrometastatic or other recurrence.

Secondary endpoints relating to short-term tolerability and toxicity will include pre-surgical treatment-related death, post-operative mortality within 30 and 90 days of surgery, completion of planned CRT, reasons for non-completion of CRT, time to adjuvant treatment after surgical resection, completion of adjuvant therapy, and frequency and severity of adverse events, including grade 4 lymphopenia. Adverse events will be graded according to CTCAE v5.0.

Secondary endpoints relating to longer-term morbidity will include total toxicity burden up to 12 months after receipt of CRT and QoL, measured using the EORTC QLQ-C30, EORTC QLQ-OES18 and EQ-5D-5 L questionnaires.

Data on health system, patient and family resource use and costs will also be collected to inform the health economic analyses.

### Sample size

It is estimated that 116 patients with OAC, 58 per arm, will be sufficient to detect a reduction in the grade ≥ 3 post-operative complication rate from 44% to 29%, with 80% power and a one-sided statistical significance level of 20%. Allowing for a 10% dropout rate, up to 130 patients with OAC will be recruited, 65 per arm. An additional cohort of 40 patients with OSCC, 20 per arm, will be included for descriptive and exploratory analysis. The total planned sample size is therefore 170 participants, comprising 85 participants per treatment arm.

### Statistical analyses

The primary analysis will compare the proportion of patients with grade ≥ 3 post-operative complications between treatment arms. The treatment effect will be estimated as the absolute between-arm difference in proportions, with 80% and 95% confidence intervals and a p-value reported. A logistic regression model adjusted for the randomisation stratification factors will also be fitted. Any additional baseline covariates included in adjusted analyses will be prespecified in the statistical analysis plan. The primary analysis population will include randomised patients who undergo surgery, analysed according to their allocated treatment arm. Death within 90 days of surgery will be counted as a post-operative complication. Patients who do not proceed to surgery, and the reasons for this, will be summarised by treatment arm. A per-protocol analysis will be performed as a sensitivity analysis. Missing primary endpoint data are not expected; if present, sensitivity analyses may be undertaken.

Secondary endpoints will be analysed using prespecified methods and according to randomised treatment allocation unless a different analysis population is prespecified. Pathological complete response and R0 resection will be summarised using frequencies and percentages among patients who undergo surgery and have evaluable pathological data. Disease-free survival and overall survival will be analysed using Kaplan-Meier estimates and Cox regression models, with medians and 95% confidence intervals reported where estimable. Time from completion of neoadjuvant CRT to surgery will be analysed using a Fine-Gray competing risks model, with disease progression, death or other reasons for not proceeding to surgery treated as competing events.

Pre-surgery toxicity-related death, suitability for adjuvant SACT, time from surgery to first adjuvant SACT, completion of adjuvant SACT, completion of radiotherapy, reasons for radiotherapy non-completion, recurrence patterns and total toxicity burden over 12 months post-CRT will be summarised by treatment arm, with between-group comparisons made where appropriate. Quality of life outcomes will be scored according to the relevant instrument manuals and summarised at baseline and follow-up timepoints.

AEs will be graded per CTCAE v5.0. The worst recorded grade for each AE type will be summarised. Frequencies and percentages of AEs by severity will be reported, including pre-surgery AEs and grade 3–5 AEs. AEs of relevance to the PROTIEUS trial will be reported by type and severity (grade 1–2 and grade ≥ 3). Grade 4 lymphopenia (CTCAE v5.0 criteria) will be summarised among participants who begin CRT.

An interim analysis will be conducted once approximately 40–50 patients have been randomised to the main study. An exact test will be performed to compare the toxicity rate to the hypothesised rate of 45%. If the toxicity rate is significantly higher or lower than the expected rate (45%) in the photon arm, then remaining SCC trial patient slots could be reallocated to adenocarcinoma.

Missing secondary endpoint data will not be imputed unless specified in the statistical analysis plan; sensitivity analyses may be undertaken where the extent or pattern of missing data could materially affect interpretation. Analyses of secondary endpoints will be exploratory, with no formal adjustment for multiplicity.

### Translational studies

Further translational analysis will be undertaken utilising blood and tissue samples and imaging studies obtained from participants. At the time of trial entry, all patients with OAC will be asked for their consent to donate archival tissue blocks and blood samples for exploratory translational research. Patients who decline can still take part in the PROTIEUS study. All patients with OAC will be invited to provide blood and tissue samples as follows:

-  Pre-treatment diagnostic biopsy tissue (including one or more formalin-fixed paraffin embedded blocks of biopsy tissue which includes the tumour.
- Blood sample for germline DNA (3mls) will be collected in an EDTA tube, pre-treatment (within 21 days prior to starting chemotherapy). This sample must not be collected before randomisation. Samples must not be frozen, but may be put into the fridge, and must be shipped on the same day as collection.
-  Blood samples for cfDNA (20mls) will be collected in STRECK tubes at the following timepoints: pre-treatment (within 21 days prior to starting chemotherapy be collected after randomisation), cycle 2 of chemotherapy, pre-chemoradiation (within 7 days prior to chemoradiation commencing), completion of CRT assessment, follow-up months 1, 3, 6, 9, 12, completion of IO assessment and disease recurrence if applicable.
- Surgical sample tissue (any residual tumour FFPE tissue blocks and one FFPE tissue block of normal mucosa, for slides).

These data will be utilised for assessment of the predictive value of pre-treatment adenocarcinoma transcriptome on response to proton based chemoradiation. Patients accrued onto trial will have circulating tumour DNA monitored as part of the follow up schema to verify the utility of ctDNA as a predictive and / or prognostic marker to inform future practice and research.

CT/PET/MRI images together with the imaging reports, will be uploaded for patients from each centre from all defined time-points: Baseline (pre-randomisation), Completion of CRT treatment, 3,6 and 12-month post-surgery follow-up assessment, Completion of immunotherapy, Disease recurrence (if applicable) and/ or any additional scans taken due to severe (grade 3) toxicity, (e.g. lung toxicity, heart failure, severe infection) (if applicable).

These imaging datasets will be analysed as part of planned sub studies for:

1. Prediction of primary endpoint (90-day surgical complications) using full spectrum multiomic data, including clinical, genetic, pathology and imaging data incorporated into hybridised deep learning models. This endpoint can serve as a basis for model development. Prediction of secondary endpoints including disease control, survival, toxicity and quality of life metrics using iterative development and retraining of the developed integrated multiomic deep learning model.
2. Imaging data will also be utilised to identify dose sensitive regions within the heart that correlate with mortality and/or cardiac toxicity in the setting of NA-IMRT and PBT for oesophageal cancer.
3. Assessment of the prognostic and predictive impact of muscle mass on clinical outcomes in patients receiving neoadjuvant chemoradiation in oesophageal cancer will be made.

## Discussion

Over the past two decades, developments in peri-operative therapy have driven considerable improvements in outcomes for patients with locally advanced OAC and OSCC [26]. Survival rates nevertheless continue to fall behind those of other cancers due to poor local control and a high rate of systemic recurrence. There is also evidence that current treatment approaches are associated with substantial morbidity. This includes short and long-term toxicities of photon-based radiotherapy in addition to the long-term reduction in health-related QoL associated with surgical resection [27]. Given this, there is a need to improve survival outcomes whilst also reducing the short- and long-term adverse impacts of therapy.

Survival can be optimised by improving non-surgical treatment approaches. In OAC, efforts to achieve this are complicated by consistent evidence across multiple trials that demonstrates up to 50% of patients are not sufficiently fit or well enough for further therapy following an oesophagectomy [5, 28]. There is therefore a need to intensify pre-operative treatment. However, peri-operative ICI has led to only modest improvements in survival in OAC, with limited evidence for an improvement in local tumour control. In addition, both OAC and OSCC are characterised by substantial inter- and intra-tumoural heterogeneity, which means there is a paucity of recurrent targets against which to target precision and personalised therapies [12, 29, 30].

These facts together suggest a continued role for radiotherapy in OAC, not least given that it has proven activity in this context. In ESOPEC, for instance, a lower pCR was seen following neoadjuvant CRT when compared with pre-operative FLOT (10.1% vs. 16.7%), yet CRT delivered > 50% tumour regression in 81.0% of patients, compared with 68.2% of patients following FLOT [5]. Further, the TOPGEAR and CRITICS-II trials demonstrated activity of CRT in gastroesophageal adenocarcinoma populations that appears additional to that of cytotoxic chemotherapy [14, 15]. Furthermore, the surgical challenges associated with an oesophagectomy are different to those of gastric cancer and may favour enhanced local control. Radiation to the stomach is also hindered by organ mobility, which is far less of an issue for tumours of the oesophagus and GOJ. This is reflected in subgroup analyses for TOPGEAR, which demonstrated improved outcomes in patients with GOJ tumours. Understanding the pathological responses following sequential SACT and hypofractionated CRT may therefore allow a move towards a surgery-as-required approach. This need is reflected by a growing number of studies focussed on combining intensified neoadjuvant systemic therapy regimes (i.e. FLOT) with CRT in OAC [31].

It is nevertheless important to reduce the morbidity associated with the use of CRT in the neoadjuvant setting, both for OAC and OSCC. By mandating daily image guidance, 4DCT and modern planning techniques (including improved dose constraints compared with the recent SCOPE-2 trial), PROTIEUS delivers contemporary radiotherapy in both treatment arms with the aim of reducing toxicity [32]. This is coupled with a comprehensive RTQA programme, which includes pre-trial benchmarking and on-trial prospective reviews to ensure protocol adherence and the delivery of high-quality radiotherapy to all patients [32]. The use of a hypofractionated approach reduces the overall treatment time and may increase efficacy given the low α/β ratios reported for oesophageal cancer [17, 18]. There is encouraging evidence of low cardiac and lung toxicity associated with the use of PBT [33]. Reduced integral dose with PBT may additionally reduce rates of grade 4 lymphopenia, which is desirable in the context of broadening use of concurrent immunotherapy schedules [20]. These early phase studies are supported by modelling that demonstrates a substantial reduction in heart and lung doses with PBT [34–38]. Using scans from the oesophageal radiotherapy-focussed SCOPE-1 trial, we have for instance shown that, when compared to photon VMAT, PBT results in a mean reduction in lung dose of 51.4% (35.1–76.1%) and heart dose of 40.9% (15.0-57.4%) [39]. PBT appears to confer a reduction in bone marrow dose, which may again have advantages for patients managed with ICI [40].

Given these promising data, an assessment of whether PBT improves treatment-related morbidity is warranted. The PROTIEUS study will address this using a protocol that allows for the provision of best systemic therapy and that delivers a shorter hypofractionated regimen of CRT that is likely to be less burdensome for patients whilst additionally benefitting from a rigorous programme of RTQA and surgical quality assurance. Importantly, this approach has been co-designed with patients with incorporation of patient views from early in trial development [41]. The data generated by this trial will complement the ongoing PROTECT and NRG GI006 trials, which are summarised in Table 1.

**Table 1:** A comparison of the PROTIEUS study and ongoing PROTECT and NRG GI006 proton trials

	UK (*PROTIEUS*)	EU (PROTECT)	USA (NRG GI006)
Status	recruiting Q4 2024	Recruiting Q2 2022	NCI recruiting Q2 2019
Trial stage	Phase 2	Phase 3	Phase 3
Eligibility	OAC and OSCC Preoperative only	OAC and OSCC Preoperative (and definitive 10%)	OAC and OSCC Preoperative and definitive
Target number	130 (OAC) 40 (OSCC)	397 (OAC and OSCC)	300 (OAC and OSCC)
IMRT arm (control)	40.05 Gy in 15 F	41.4 Gy in 23 F (60%) 50.4 Gy in 28 F (40%)	50.4 Gy in 28 F
Proton arm	40.05 Gy in 15 F (RBE)	41.4GyE in 23 F (60%) 50.4GyE in 28 F (40%)	50.4 Gy in 28 F
Chemotherapy	Carbo-taxol x5C OR FLOT x 4 (neoadjuvant) +/- IO, carbo-taxol x 3, FLOT x 4 (adjuvant) +/- IO	Carbo-taxol x5C (or 6 C in 50.4 Gy arm)	Carbo-taxolx6 or FOLFOX
Centres	15 UK (2 proton centres new technology)	Proton centres (new and old technology) in 9 European countries	US proton centres (new and old technology)
Primary aim & endpoint for PBT	Improvement in 90-day grade 3–5 toxicity	Improvement in 90-day post-surgery or definitive CRT pulmonary complications	OS to be non-inferior (or superior) with less cardiopulmonary toxicity

*C* Cycles, *Carbo-taxol* Carboplatin/paclitaxel, *CRT* Chemoradiotherapy, *F* Fractions, *FLOT* 5-fluorouracil, leucovorin, oxaliplatin, docetaxel, *FOLFOX* 5-fluorouracil, leuvocorin, oxaliplatin, *Gy* Gray, *GyE* Gray equivalent, *IO* Immunotherapy, *OAC* Oesophageal adenocarcinoma, *OS* Overall survival, *OSCC* Oesophageal squamous cell carcinoma, *Q* quarter, *RBE* Relative biological effectiveness

The PROTIEUS trial is a prospective investigator-led multi-centre phase 2 study that will compare photon- versus PBT-based neoadjuvant CRT following best peri-operative systemic therapy in patients with locally advanced OAC and OSCC. As interest in total neoadjuvant approaches grows, this study will determine the potential for incorporation of hypofractionated radiotherapy into neoadjuvant regimens in oesophageal cancer and determine the optimal approach for delivering radiation in this context. Results from this trial will be communicated via peer review publications and plain language summaries upon completion, as per the trial publication policy. 

## Supplementary Information

Supplementary Material 1.

Supplementary Material 2: Supplementary file 1: Example schedules for proton- and photon-based treatment in OAC and OSCC. Supplementary file 2: PROTIEUS Radiotherapy Planning Guidance Document. Supplementary File 3: Schedule of events. ^A^ Contrast enhanced. Neck optional. ^B^ Weight should be recorded at every visit where indicated, with addition of height to be recorded at screening only. ^C^ Full physical examination to be completed at screening and C1 neoadjuvant chemotherapy treatment, and all visits thereafter should be targeted physical assessment only. ^D^ Vital signs should include blood pressure, temperature, heart rate. ^E^ Not required if CPEX/CPET done (not mandatory but strongly recommended). ^F^ Weekly assessment should be done within 72 hours of the start of each chemotherapy cycle. ^G^ Required only if clinically indicated. ^H^ Pregnancy test does not need to be repeated if screening test was performed within 7 days of starting CRT. ^J^ Must be carried out after randomisation and prior to any chemotherapy delivery. ^K^ Diagnostic and surgical FFPE samples to be sent to central lab for exploratory research. ^L^ Pulmonary function test (e.g. cardiopulmonary exercise testing such as CPEX/CPET or lung function tests) (not mandatory but strongly recommended). ^M^ Patients that do not have surgery will be followed up as per local policy. Disease recurrence and death should be reported. EQ-5D-5L to be completed. ^N^ Other imaging as per local policy. ^P^ Whole Body PET-CT and CT are both required prior to randomisation, however, only 1 of these scans must be performed within 4-6 weeks prior to randomisation. ^Q^ Cardiopulmonary function testing must be performed within 12 weeks pre-randomisation (e.g. ECG and MUGA or ECHO) according to local policy. If it is not possible to complete test pre-randomisation, test must be performed before treatment starts. At completion of CRT treatment and follow-up only ECG is required. R CT and/or PET-CT required at 6 and 12 month follow-up visits. ^T^ EUS is strongly recommended within 6 weeks prior to randomisation, (but not mandatory) and laparoscopy is strongly recommended within 6 weeks prior to randomisation. Laparoscopy should be performed as per institutional standard of care for GOJ tumours. ^U^ Full blood count and differential: Haemoglobin, white blood cell count, platelet count, absolute neutrophil count, absolute lymphocyte count. ^V^ Serum biochemistry, renal and liver function: albumin, lactate dehydrogenase (LDH), urea, creatinine, sodium, potassium, bilirubin, aspartate transaminase (AST) and/or alanine transaminase (ALT). SACT= systemic anticancer therapy. * Tests required before randomisation but no specific time constraint. CSRI = Client Service Receipt Inventory.

## Data Availability

No datasets were generated or analysed during the current study.

## Appendix

### Indemnity

University College London holds insurance against claims from participants for injury caused by their participation in the clinical trial. Participants may be able to claim compensation if it is proven that UCL has been negligent. However, as this clinical trial is being carried out in a hospital, the hospital continues to have a duty of care to the participant of the clinical trial. University College London does not accept liability for any breach in the hospital’s duty of care, or any negligence on the part of hospital employees. This applies whether the hospital is an NHS Trust or otherwise.

Participants may also be able to claim compensation for injury caused by participation in this clinical trial without the need to prove negligence on the part of University College London or another party. Participants who sustain injury and wish to make a claim for compensation should be advised to do so in writing in the first instance to the Chief Investigator, who will pass the claim to the Sponsor’s Insurers, via the Sponsor’s office.

Hospitals selected to participate in this clinical trial shall provide clinical negligence insurance cover for harm caused by their employees and a copy of the relevant insurance policy or summary shall be provided to University College London, upon request.

## Abbreviations

CRT: Chemoradiotherapy
CT: Computed tomography
FLOT: 5-fluorouracil with leucovrin, oxaliplatin and docetaxel
GOJ: Gastroesophageal junction
OAC: Oesophageal adenocarcinoma
OSCC: Oesophageal squamous cell carcinoma
PBT: Proton beam therapy
PET: Positron emission tomography
pCR: Pathological complete response
QoL: Quality of Life
