# Supplementary material for: Pre-operative proton versus photon-based chemoradiotherapy as an addition to best systemic therapy in the management of oesophageal cancer: protocol for the UK multi-centre randomised phase 2 PROTIEUS study
Source: BMC Cancer. 2026 Jun 29;26:1101. doi: 10.1186/s12885-026-16424-1 (PMC13579814; doi:10.1186/s12885-026-16424-1)
Supplement: Supplementary file 2 [file 12885_2026_16424_MOESM2_ESM.docx]

**SUPPLEMENTARY MATERIALS**

**Supplementary file 1: Example schedules for proton- and photon-based treatment in OAC and OSCC.**

**OAC**

**OSCC**

**Supplementary file 2: PROTIEUS Radiotherapy Planning Guidance Document**

**Supplementary File 3: Schedule of events.** ^A^ Contrast enhanced. Neck optional. ^B^ Weight should be recorded at every visit where indicated, with addition of height to be recorded at screening only. ^C^ Full physical examination to be completed at screening and C1 neoadjuvant chemotherapy treatment, and all visits thereafter should be targeted physical assessment only. ^D^ Vital signs should include blood pressure, temperature, heart rate. ^E^ Not required if CPEX/CPET done (not mandatory but strongly recommended). ^F^ Weekly assessment should be done within 72 hours of the start of each chemotherapy cycle. ^G^ Required only if clinically indicated. ^H^ Pregnancy test does not need to be repeated if screening test was performed within 7 days of starting CRT. ^J^ Must be carried out after randomisation and prior to any chemotherapy delivery. ^K^ Diagnostic and surgical FFPE samples to be sent to central lab for exploratory research. ^L^ Pulmonary function test (e.g. cardiopulmonary exercise testing such as CPEX/CPET or lung function tests) (not mandatory but strongly recommended). ^M^ Patients that do not have surgery will be followed up as per local policy. Disease recurrence and death should be reported. EQ-5D-5L to be completed. ^N^ Other imaging as per local policy. ^P^ Whole Body PET-CT and CT are both required prior to randomisation, however, only 1 of these scans must be performed within 4-6 weeks prior to randomisation. ^Q^ Cardiopulmonary function testing must be performed within 12 weeks pre-randomisation (e.g. ECG and MUGA or ECHO) according to local policy. If it is not possible to complete test pre-randomisation, test must be performed before treatment starts. At completion of CRT treatment and follow-up only ECG is required. ^R^ CT and/or PET-CT required at 6 and 12 month follow-up visits. ^T^ EUS is strongly recommended within 6 weeks prior to randomisation, (but not mandatory) and laparoscopy is strongly recommended within 6 weeks prior to randomisation. Laparoscopy should be performed as per institutional standard of care for GOJ tumours. ^U^ Full blood count and differential: Haemoglobin, white blood cell count, platelet count, absolute neutrophil count, absolute lymphocyte count. ^V^ Serum biochemistry, renal and liver function: albumin, lactate dehydrogenase (LDH), urea, creatinine, sodium, potassium, bilirubin, aspartate transaminase (AST) and/or alanine transaminase (ALT). SACT= systemic anticancer therapy. * Tests required before randomisation but no specific time constraint. CSRI = Client Service Receipt Inventory.

| **Timepoint** | Pre-randomisation | Pre-Neoadjuvant  SACT | During Neoadjuvant SACT | During concurrent Chemoradiation (CRT)^F^ | Completion of CRT | **Surgery^M^** | **Adjuvant SACT**  (2-weekly) (within 4- 12 wks post-surgery) | Post Surgery Follow-Up  Month 1 | Post Surgery Follow Up  Month 3 **(PRIMARY ENDPOINT)** | Post Surgery Follow Up  Months 6, 9 &12 | Completion of SACT (including after 1 yr administration, unacceptable AE, IO withdrawal) | Disease Recurrence |
| --- | --- | --- | --- | --- | --- | --- | --- | --- | --- | --- | --- | --- |
|  | Within 7d pre-randomisation (*unless indicated) | Within 7d prior to starting neoadjuvant SACT | FLOT+/-IO:  2-weekly before cycles 2,3,4  AND week prior to chemoradiation  Carbo-taxol: weekly | Chemo: 3 weekly cycles,  RT/PBT over 3 consecutive weeks | 4-6 wks post last fraction |  |  | 4 wks post surgery (+/- 14days) | 13 wks post surgery (+/- 7days) | 6, 9 & 12 mths post surgery (+/- 14days) | 28-35d post last SACT administration |  |
| Informed Consent* | x |  |  |  |  |  |  |  |  |  |  |  |
| Histology confirmation of OEC^K*^ | x |  |  |  |  | x |  |  |  |  |  |  |
| Upper GI MDT Confirmation of eligibility | X* |  |  |  |  |  |  |  |  |  |  |  |
| DPYD test | X* |  |  |  |  |  |  |  |  |  |  |  |
| Whole body PET-CT | x^P^ |  |  |  | x^G^ |  |  |  |  | x ^R^  (6&12m only) |  |  |
| CT (TAP)^A^ | x^P^ |  |  |  | x |  |  |  | x | x ^R^  (6&12m only) | x | x^N^ |
| EUS (recommended) and Laparoscopy (GOJ tumours only) | x^T^ |  |  |  |  |  |  |  |  |  |  |  |
| Medical History | x |  |  |  |  |  |  |  |  |  |  |  |
| Assessment of AEs | x |  | x | x | x |  | x | x | x | x | x | x |
| Physical examination^C^ | x |  | x | x | x |  | x | x | x |  |  |  |
| Weight ^B^ | x^B^ |  | x | x |  |  | x |  |  |  |  |  |
| Vital signs^D^ | x |  |  |  |  |  |  | x | x |  |  |  |
| Mellow-Pinkas dysphagia score | x |  | x | x | x |  |  |  |  |  |  |  |
| ECOG PS | x |  | x | x | x |  | x | x | x | x (6m only) |  |  |
| Cardiac function testing^Q^ | x^Q^ |  |  |  | x |  |  | x | x | x |  |  |
| Pulmonary function test ^Q^ | x^Q^ |  |  |  | x^E,L^ |  |  | x^E,L^ | x^E,L^ | x^E,L^ |  |  |
| FBC + differential^U^ | x |  | x | x | x |  | x | x | x | x | x |  |
| Serum biochemistry, renal and liver function^V^ | x |  |  | x |  |  |  |  |  |  |  |  |
| Pregnancy test | x | x^H^ |  |  |  |  |  |  |  |  |  |  |
| QoL  (EORTC QLQ-C30 +  QLQ-OES18) |  | x |  |  |  |  |  | x | x | x |  | x |
| QoL  (EQ-5D-5L) |  | x | x (week before CRT only) |  | x |  |  | x | x | x |  |  |
| Participant Health Care Resource Use Questionnaire (Modified CSRI) |  | x |  |  | x |  |  | x | x | x |  |  |
| Post-operative complications (Clavien-Dindo Appendix 4) |  |  |  |  |  |  |  | x | x |  |  |  |
| Research blood (cfDNA) |  | x and germline^J^ | x (in the week before CRT only) |  | x |  |  | x | x | x | x | x |
| Confirmation of commencement of immunotherapy treatment |  |  |  |  |  |  |  | x |  |  |  |  |
| Review of further/additional treatment |  |  |  |  |  |  |  |  |  |  |  | x |
